# An Exceptional Case of Liver-Restricted High-Grade B-Cell Lymphoma in a Patient with Clinical History of HBV and HCV Coinfections

**DOI:** 10.1155/2019/5125086

**Published:** 2019-02-14

**Authors:** Connor R. Zuraski, Vinit Patil, Katherine E. Schwetye

**Affiliations:** Department of Pathology, Saint Louis University School of Medicine, 1402 South Grand Boulevard, Saint Louis, MO 63110, USA

## Abstract

Primary hepatic lymphomas (PHLs) are exceedingly rare. Many reported cases are associated with various viral serologies, and some viruses may be implicated in lymphomagenesis through emerging, though as-of-yet uncertain, mechanisms. A review of the literature reveals previously reported cases of PHL, some of which support the potential roles of the hepatitis B and C viruses (HBV and HCV) in the development of PHL. We describe an exceptional case of primary hepatic high-grade B-cell lymphoma, discovered at autopsy, in a patient whose clinical history is significant for coinfection with both HBV and HCV. Additionally, attempts at cytogenetic testing of formalin-fixed, paraffin-embedded (FFPE) autopsy tissues, which we performed approximately ten years after the original autopsy, led us to question the utility of older tissue blocks in molecular and some immunohistochemical assays.

## 1. Introduction

Primary hepatic lymphomas are unusual entities: primary hepatic non-Hodgkin lymphomas (NHLs) comprise 0.4% of all primary extranodal lymphomas and 0.016% of all NHLs [1]. Primary hepatic high-grade B-cell lymphoma is rarer still. Approximately 95% of liver-restricted NHL cases are diffuse large B-cell-type, and the remaining 5% include Burkitt-type and other high-grade morphologies [1]. Many of these reported cases are associated with diverse viral serologies, which commonly include HBV or HCV. The relationship between hepatitis B infection and primary NHL is not strongly established; some sources state that no statistically significant relationship between the two may exist at all [1]. However, transcriptomics research has identified pathways leading to the development of HBV-associated diffuse large B-cell lymphomas (DLBCLs) [2]. Concerning HCV, there is an association between HCV infection and mixed cryoglobulinemia type II (MC), which significantly implicates HCV in the clonal B-cell expansion that leads to MC [3]; thus, it may explain the appearance of NHL in some individuals. We describe the second reported case of primary hepatic high-grade B-cell lymphoma, discovered at autopsy, in a patient previously coinfected with both HBV and HCV.

## 2. Case Presentation

We performed a search and systematic review of all autopsy reports in the electronic database at Saint Louis University Hospital between 1996 and 2016 for the term “lymphoma”. The inventory of discovered cases was then categorized based on the number and type of organs affected by the lymphoma, concomitant neoplastic processes, association with distinct infections, and unique and potentially intriguing cases. One case of primary hepatic lymphoma was identified in a 55-year-old man with a known history of HBV and HCV infections as well as cirrhosis; he had not previously been treated with antiviral therapies. No prior laboratory testing results were identified in our hospital system. The patient presented suddenly to the emergency department with mental status changes attributed to hepatic encephalopathy. Endoscopy revealed grade II esophageal varices and a flat-based ulcer near the gastroesophageal junction. The patient eventually developed multiorgan failure with coagulopathy and passed away. Death was due to liver failure in the setting of cirrhosis.

At the 2007 autopsy, the 2150-gram liver was extensively nodular, including areas of central necrotic parenchyma surrounded by a hemorrhagic rim. A focal area of gray-white parenchyma with an infiltrative appearance obscuring the cirrhotic nodules was determined to be probable Burkitt lymphoma by histomorphologic and limited immunohistochemical evaluation per the attending pathologists and according to the WHO classification at that time. Due to the infiltrative nature of the lesion, accurate gross dimensions proved difficult to measure. Microscopically, the cells in these areas were arranged in large nodules and were intermediate in size and mitotically active. The nodules were associated with necrosis and numerous apoptotic bodies. Extensive assessment revealed no other organs or lymphoid tissues to be involved by lymphoma, including the brain. Notably, sections of the left ventricle showed patchy subendocardial coagulative necrosis with a few polymorphonuclear cells, indicating an acute infarct one to several days old.

Almost ten years after the original autopsy, we ordered additional recut sections of the FFPE liver tissue blocks for potential cytogenetic testing. The following stains were performed on recut sections: hematoxylin and eosin (H&E), CD5, CD20, CD10, BCL-6, BCL-2, Ki-67 (MIB-1), Gömöri trichrome, and Epstein–Barr virus in situ hybridization (EBV-ISH). In preparation for fluorescence in situ hybridization (FISH), 4-micron-thick sections were cut, floated on a purified (*i.e.,* triple-distilled) water bath at 40°C, mounted on a positively charged slide, and air dried.

**Figure 1:**
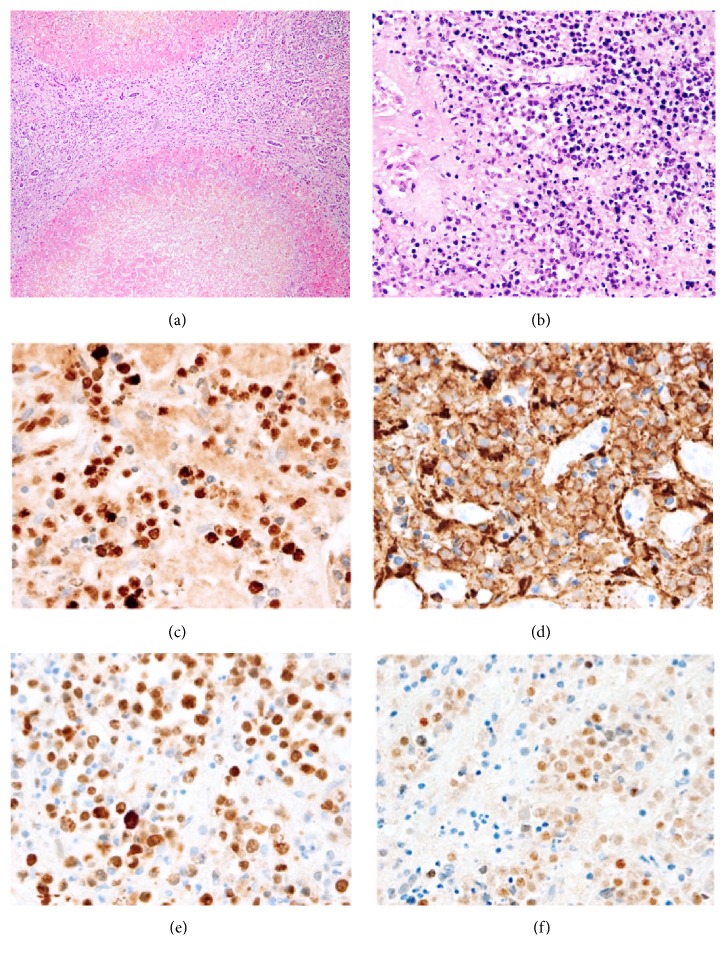
Broad fibrous bands separate nodules of necrotic hepatic parenchyma in the setting of cirrhosis (a) and diffuse infiltration of hepatic parenchyma by lymphoma cells admixed with nonneoplastic lymphocytes (b) (H&E); CD10 (c), CD20 (d), and BCL-6 (e) immunostains are strongly and diffusely positive in lymphoma cells; Ki-67 (f) shows a high proliferative index of nearly 100% in malignant cells, which are admixed with nonneoplastic lymphocytes.

Recut H&E- and Gömöri trichrome-stained sections of liver showed the same nodules of autolyzed and necrotic hepatic parenchyma with intervening fibrous bands that were described in the original report (Figure 1(a)). There were also nodular areas of diffusely infiltrative, intermediate-sized lymphoid cells (Figure 1(b)). Evaluation of immunohistochemical stains (IHCs) performed after the original autopsy in 2007 and on the original tissue block recuts again in 2016 is summarized as follows: positive for CD20, CD10, and BCL-6 and negative for CD5, BCL-2, cyclin D1, and EBV-ISH. Ki-67 immunostaining shows a high proliferative index in this cell population. The interpretations in the original autopsy report are in accordance with ours after we performed selected immunohistochemistry on the recut sections (selected IHC micrographs appear in Figures 1(c)–1(f)).

## 3. Discussion

Our results from this case describe a primary hepatic high-grade B-cell lymphoma associated with a remarkable combination of HBV and HCV infection, which has been reported only one other time in the literature [3]. Since no lymph node or marrow involvement was identified in our case, the entity is likely to be a primary hepatic lymphoma. Accordingly, the interaction of these two viruses likely played an important role in lymphomagenesis within the liver itself by the mechanism of signaling interference. The possibility that this high-grade PHL arose from a low-grade lymphoma cannot be entire ruled out [3].

Interestingly, both HBV and HCV are capable of disrupting multiple molecular pathways and the normal cellular microenvironment in the liver [4]. The alterations are conducive to the development of some neoplasms, including lymphomas. However, the exact mechanisms through which these changes play a role in the process of malignant transformation or definite activation of viral oncogenes have not yet been fully clarified. A study of French patients with primary hepatic NHL indicates that chronic B-cell stimulation by external viral antigens causes incessant proliferation, which may ultimately result in monoclonal expansion [5]. The molecular target has been identified: it has previously been shown and the findings replicated that CD81 acts as a receptor on B cells for the HCV virus; CD81 also contributes to tumor growth and metastasis in a variety of malignancies [6]. Though HCV does not integrate into the host genome [7], viral proteins may exert an oncogenic effect on B cells as the virus replicates inside them. Temporary intracellular presence of the virus may also contribute to irreversible mutations in tumor suppressor genes [8] and transcriptional changes of regulatory host genes mediated by HCV-associated proteins [5]. Furthermore, HCV may drive the induction of the t(14;18) translocation, which results in overexpression of BCL-2 and rearrangement of monoclonal immunoglobulin heavy chain (IgH), although this mechanism is thought to occur more frequently in cases of patients with mixed cryoglobulinemia (MC) [5].

The role of HBV in the development of PHL is not well understood, although there is surfacing evidence regarding the association of HBV with DLBCL. Meta-analysis of observational studies has shown that HBV, like HCV, is associated with a two- to threefold risk of NHL [2, 8]. Long-term clinical data from one study of patients in China reveal that the rate of positive serum HBV was increased significantly in DLBCL patients (23.6%) compared to the rate in the general Chinese population (7.2%) [9]. In another review of 69 individuals who developed primary hepatic lymphoma, 20% were positive for HBV surface antigen (HBsAg) [10]. In HBsAg-positive patients, the intracellular presence of the HBV antigen and HBV-specific nucleic acid sequences supports the theory that HBV may play a significant role in lymphomagenesis [9]. HBV-encoded X protein (HBx) is capable of p53 inhibition [2], a mechanism similar to that observed in the malignant transformation of hepatocytes [9], leading to hepatocellular carcinoma, and may also contribute to the development of B-cell NHL. Moreover, among patients with DLBCL, HBV infection was associated with younger age (42 versus 60 years old) and more advanced disease stage at diagnosis [2]. Transcriptome sequencing showed unique gene expression profiles in tumors positive for HBsAg, including upregulation of p53 signaling and antigen processing and presentation pathways [2]. Finally, through the examination of IgH sequences, researchers suggest that an antigen-independent process, as opposed to one based on chronic antigenic stimulation, is the probable mechanism in HBV-related lymphomagenesis [2].

Still more curious is the discussion of whether* coinfection* with both HBV and HCV might uniquely affect the induction, progression, or overall clinical prognosis of high-grade B-cell lymphomas. Only one other reported case of such a coinfection in a patient who developed primary hepatic high-grade B-cell lymphoma currently exists in the literature. The patient was a 75-year-old man with known chronic hepatitis C and resolved hepatitis B infections, both of which were diagnosed after blood transfusion following a car accident nearly thirty years before autopsy [3]. His clinical history did not include MC [3]. Unlike the patient at our institution, this man's high-grade B-cell lymphoma was diagnosed on biopsy following a clinical presentation suggestive of malignancy [3]. Extensive workup revealed no splenomegaly or pathological lymphadenopathy identified by computed tomography, although chest X-ray did reveal irregularities suspicious for metastases to both lungs [3]. Though he received appropriate polychemotherapy and entered complete remission over a three-year period, the patient ultimately passed away following myeloid blast crisis [3]. Despite these two patients' strikingly different clinical courses, they share a history of a unique combination of viral serologies in the setting of primary hepatic high-grade B-cell lymphoma. Certainly, reactivation of latent infection in association with chemotherapy and immunosuppressive management is a well-known phenomenon and likely played a role in the clinical course and death of the above-described patient [3], whereas our patient died without clinical knowledge or suspicion of disease.

Molecular testing is frequently limited in archival FFPE tissue, particularly with respect to analysis of ribonucleic acid (RNA). RNA degrades in cells prior to fixation in formalin, moreso during the postmortem interval prior to autopsy than during the so-called “cold ischemic” time in surgical specimens [11]. Formalin alters RNA, and even after the process of paraffin embedding, RNA molecules continue to degrade over time [11]. A 2007 study considered the qualitative and quantitative potential for RNA extraction from ten-year-old FFPE tissue blocks and three- to ten-month-old blocks using four protocols, including spin column purification and magnetic bead-based techniques; the size of retrievable RNA fragments differed between the months-old and ten-year-old tissues, with ~151 bp as the upper limit of retrievable material in the older tissues [11].

Given the 10-year age of the archival FFPE tissue blocks in our case, FISH was attempted using a dual-labeled break-apart probe directed onto* C-MYC* at the 8q24 locus to detect a potential translocation. The procedure using regular protocol yielded no signal, and repetition using a double-treated slide yielded normal signal in only 5 interphase cells. Because a minimum of 100 cells is required to report a finding, this assay was technically limited. Immunophenotypically, the lymphoma in our case fits the profile of Burkitt-type. However, because FISH did not elucidate the cytogenetic aspects of the tumor, the broader diagnosis of high-grade B-cell lymphoma is most appropriate according to the most current WHO criteria [12].

In situ hybridization for Epstein–Barr virus on liver sections was evaluated as negative in our case. However, this result should be interpreted with caution. Depending on the staining technique employed, the sensitivity for detection of viral RNA in archival FFPE tissue may be significantly lower than when the same procedure is performed on nonarchival tissue.

The above-described limitations raise a serious issue in the follow-up of patients. Although our described case was that of an autopsy specimen, it is critically important to be able to perform further analyses on archival tissue from surgically resected specimens, even after they may be years-old. For example, newer therapies for breast cancer, such as Her2 monoclonal antibodies, may be appropriate for a late metastasis; the ability to test archival tissue would theoretically save a patient from an invasive, costly, painful, and potentially morbid biopsy procedure (such as a biopsy or resection of a brain metastasis requiring craniotomy). The case we describe also brings to light another issue, that of the reclassification of certain tumors as discovery of molecular markers help refine diagnoses. The ability to test archival tissues for the newer molecular markers is critical to accurately diagnose and treat patients, to move forward, and to perform retrospective research. For these reasons, alternative methods of tissue preservation need be explored, such as tissue banking, which requires resources to snap-freeze and store frozen tissues at -70 C. The balance of optimal tissue preservation and cost is a delicate one. Currently, in the United States, tissue banking frozen tissues is not required by the accrediting agency (College of American Pathology/CLIA) standards. However, as molecular diagnoses are continually and increasingly becoming standard of practice, particularly in the realm of hematopoietic disease, better preservation of archived tissues will be necessary.

In summary, the patient's autopsy confirmed the clinical diagnosis of end-stage cirrhosis and acute myocardial infarction. The contribution of the patient's liver-restricted lymphoma to his clinical course and death is unclear. Aside from its rarity, the discovery of this lymphoma at autopsy is not unduly surprising: due to its diffuse, infiltrative nature and the nonspecific symptoms with which an afflicted patient may present, radiologic and clinical diagnosis of this condition proves challenging. Immunohistochemistry and the histomorphologic appearance of the malignant cells were essential to the diagnosis of high-grade B-cell lymphoma.

In general, RNA extraction from ten-year-old FFPE autopsy tissue blocks is limited and may require multiple techniques. Molecular testing using probes directed at specific RNA sequences is also challenging and failed in this case, despite conventional and double-treated procedural attempts. Alternatives to FFPE tissue preservation, such as tissue banking at -70 C, may be the preferred method for optimizing the ability to perform future molecular testing.

Current literature suggests a potential causative role of HBV and HCV in lymphomagenesis, albeit through a variety of mechanisms. The potential roles of either virus alone, dual coinfection status, and the tissue microenvironment (*i.e.,* cirrhotic liver) are aspects of the development of primary hepatic lymphoid malignancies that are yet to be fully elucidated and require ongoing investigation and thorough documentation.
